# Death and Seeking Alternative Therapy Largely Accounted for Lost to Follow-up of Patients on ART in Northwest Ethiopia: A Community Tracking Survey

**DOI:** 10.1371/journal.pone.0059197

**Published:** 2013-03-18

**Authors:** Mamo Wubshet, Yemane Berhane, Alemayehu Worku, Yigzaw Kebede

**Affiliations:** 1 Institute of Public Health, University of Gondar, Gondar, Ethiopia; 2 Addis Continental Institute of Public Health, Addis Ababa, Ethiopia; 3 School of Public Health, Addis Ababa University, Addis Ababa, Ethiopia; Fundacion Huesped, Argentina

## Abstract

**Background:**

Antiretroviral treatment programs in sub-Saharan African countries are highly affected by LTF. Tracking patients lost to follow-up and understanding their status is essential to maintain program quality and to develop targeted interventions to prevent LTF. We aimed to determine the outcome and factors associated with LTF.

**Method:**

A lost to follow-up community tracking survey was conducted to determine the reasons, outcomes and factors associated with LTF at the University of Gondar Hospital, northwest Ethiopia. All patients were tracked at home to ascertain outcome status for lost to follow-up (death and non-death losses).

**Result:**

Out of the 551 patients LTF, 486 (88.20%) were successfully tracked. Death was the most common reason accounted for 233 (47.94%) of the lost to follow-up. Reasons for non-deaths losses include: stopped antiretroviral treatment due to different reasons, 135(53.36%), and relocation to another antiretroviral treatment program by self- transfer, 118(46.64%). The rate of mortality in the first six months was 72.12 per 100 person-years (95% CI: 61.80–84.24) but this sharply decreased after 12 months to 7.92 per 100 person-years (95% CI: 4.44–14.41). Baseline clinical characteristics were strongly associated with mortality.

**Conclusion:**

Death accounts for about half of the loss to follow up. Most deaths occur in the first six months of loss. Seeking alternative therapy is another major reason for loss to follow up. Early tracking mechanisms are necessary to prevent death.

## Background

Antiretroviral treatment (ART) programs in sub-Saharan African countries are highly affected by loss to follow-up (LTF). LTF remains one of the major challenges to the success of ART programs in these settings [1]–[3]. A systematic review of ART programs in sub-Saharan Africa found that about 40% of patients were lost in two years, with large variations in retention rates [4]. In resource-limited settings 1 in 5 of patients are lost in the first six months of initiating ART [5]. Furthermore, it is known that LTF is the most cause of attrition in such settings [6]. On the other hand, the outcome of a third of patients LTF remained unknown in such settings [7].

The outcome of patients LTF in Africa has received relatively little attention. Patients who stopped taking antiretroviral drugs are resulting in high mortality. On the other hand, with increasing availability of ART, patients may have official or self-transferred to another ART program, for example, a program closer to their place of residence. Identifying and tracking patients who are potentially LTF and understanding their status is essential to maintain program quality and to develop targeted interventions to prevent LTF and reduce mortality and thus identify practical solutions that can improve retention.

We performed a community tracking for patients LTF from the ART clinic at the UoG Hospital. The objectives of the study were to determine the reasons and outcomes of patients LTF.

## Methods

### Setting

The study was conducted in the ART Program run by the UoG Teaching Hospital, in northwest Ethiopia, described in detail elsewhere [8]. The national HIV prevalence was 1.5% in 2011 [9]. Patients from other facilities are referred to the ART program. Patients routinely pick up antiretroviral drug supplies for one month. A limited number of patients may however collect their three-month supply at a time for various reasons. TB/HIV Co-trimoxazole Preventive Therapy (CPT) is recommended to all TB/HIV co-infected patients regardless of the CD4 count [10].

Patient’s paper record is regularly transferred to the electronic database. After patients are referred by the ART clinician, Case managers are responsible for adherence counseling and managing the ART risk factors. Patients who fail to return on their designated appointments are tracked by Case managers. Initial tracking is attempted by telephone by the clinic nurses on the day following the missed appointment. If this is unsuccessful, Case managers are deployed to patients’ respective addresses. *Case managers* are certified care givers with a modest experience of community health. Patients at the ART Clinic are referred by ART clinician to Case managers for adherence and managing ART risk factors. Case managers also conduct planned outreach activities to track LTF and onsite community support. As part of the government program, the clinic provides care and support to people living with HIV free of charge according to the national and World Health Organization (WHO) guidelines [11], [12].

### Study Design and Data Source

**Figure 1:**
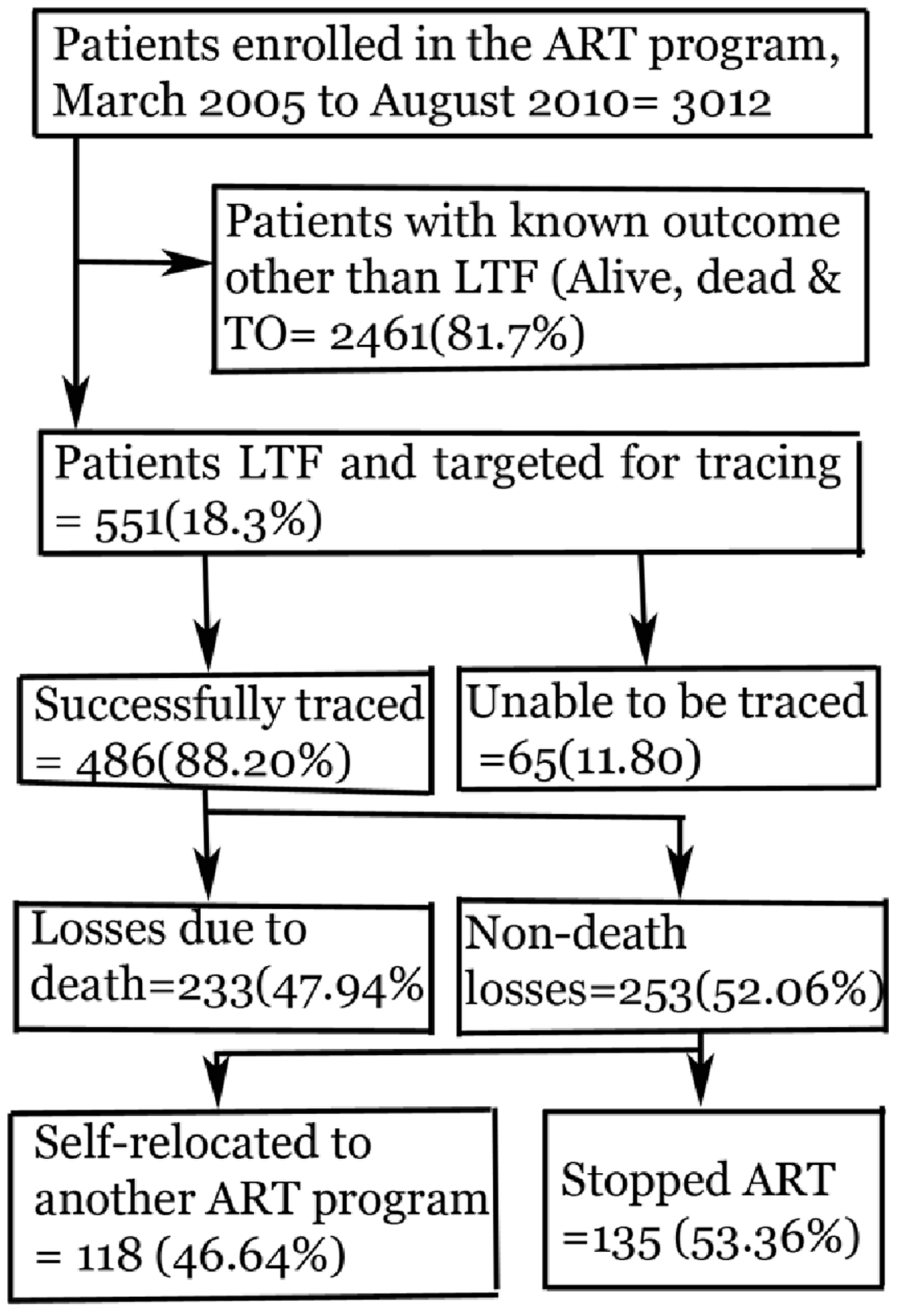
Schematic diagram showing the procedure of tracking LTF.

A community tracking survey was conducted to determine the outcome and factors associated with LTF at UoG Hospital, northwest Ethiopia. This study involved patients in the treatment program from March 2005 through August 2010 (Figure 1). Patients who missed their last scheduled visit by 3 months, as proposed by the National ART Program guidelines [11], were tracked to find out the reason and outcomes for LTF.

### Study Population and Data Collection

Between September and October 2010, we tracked adult patients who were 3 months late for their appointment to pick-up their antiretroviral drugs. The database maintained for similar purpose had contact information and baseline characteristics. Baseline clinical characteristics were taken from the patient card. Outcomes and reasons of tracked LTF were collected using a standardized semi-structured questionnaire which was translated to Amharic and back translated to English, and was piloted in the field.

Case managers contacted patients or their family members in person in their homes to determine whether the patient’s LTF was related to death or non-death. If patients were tracked at home, they were asked whether they were still taking ART and if not, why they had stopped. If the patient had died, the relatives were asked when the patient had died. If the patient had moved away, relatives or friends were asked where they had moved. The contact details of immediate family member or friend in the patient card was taken for such consultations.

*Non-death losses* were the sum total of self-transfer-outs and true losses to follow-up. *Self transfer-outs* were patients whose care was transferred to other facilities by patients themselves, unlike to facility transfer outs whose care transfer is official. *True losses* were those patients whose information was not able to be tracked by the community tracking survey. For patients who were apparently alive but stopped taking ART at the time of data collection, we asked the reasons for not returning to the clinic.

### Statistical Analysis

Data were analyzed using Stata, version 11(Stata Corp). The main outcome measures were patients who were lost to the program due to death and non-death reasons. Descriptive statistics was used to characterize the LTF. We calculated rates of mortality from the date of ART initiation and date of LTF registered in the database. Person-time was censored at the end of August 2010 for individuals who were found to be alive but stopped treatment. Deaths were censored on the date of death reported by family members. Then mortality rates were calculated by dividing deaths by person-year to get mortality rates per 100 person-years of observation.

We estimated time to LTF and death by Kaplan-Meier failure function. In other product-limit analyses, log rank tests were used to examine the effect of baseline clinical characteristics on LTF probabilities. Product limit analysis was used to calculate the instantaneous hazard of death or other losses through time among individuals receiving ART; we plotted smoothed hazard-function estimators using weighted kernel-density estimates based on the Gaussian function.

Cox-proportional hazard models were used to examine determinants of mortality and non-death losses among individuals LTF in the treatment program. Separate models were developed to examine factors associated with reasons for LTF (death and none death reasons). We considered the model which included all the predictors that had a *P* value of less than 0.20 in the univariate analyses. Covariates predetermined to be in the univariate analysis were entered to full multivariable model. Model diagnostics and the proportional hazards assumption were examined using Schoenfield residuals [13]. The following baseline covariates were entered into the model: age, sex, marital status, occupation, disclosure status, presence of tuberculosis, place of residence, functional status, WHO stage, and CD4 count.

### Ethics Statement

This study obtained ethical clearance from the Institutional Review Board of the UoG, Ethiopia. All patients successfully tracked for the study provided a written informed consent. Patients found to be alive but stopped ART, counseling was given to continue their treatment by the Case managers during data collection. Care was taken to keep all patient information confidential during tracking.

## Results

### Cohort Characteristics and Tracking Patients

Five hundred fifty-one patients were lost out of the 3012 who were initiated on ART be between March 2005 and August 2010 were used for this study (Fig. 1). Follow-up time before loss to follow up ranged from 30 to 1447 days with a median follow-up time of 302 days (IQR: 152–627). Overall patients enrolled in this study contributed 624.25 person-years of observation. Baseline socio-demographic and clinical characteristics of the patients who were LTF on ART is presented in Table 1. The median age was 33 years (IQR: 28–40). The majority (71.5%) came from outside Gondar town. Their clinical characteristics also showed that 46.5% did not disclose their HIV status, 40% had tuberculosis co-infection at ART initiation. The mean CD4 cell count was 68 cells/µl (IQR: 41–100). Baseline functional status and WHO clinical stage showed that about 51% were either ambulatory or bed ridden and 39% were WHO stage III or IV, respectively.

**Table 1:** Demographic and clinical characteristics of patients included in LTF tracking study (N = 551).

Characteristics	Subcategory	Number (%)
Gender	Male	301(54.63)
	Female	250(45.37)
Age, years	Median (IQR)	33(28–40)
	<25	57 (10.34)
	25–39	326 (59.17)
	40–54	146 (26.50)
	≥55	22(3.99)
Marital status	Currently married	229 (42)
	Currently not married	298 (54)
	Missing	22(3.99)
Educational status	Not educated	264 (47.91)
	Primary	190 (34.48)
	Secondary and above	54 (9.80)
	Missing	43 (7.80)
Employment status	Currently unemployed	262 (47.55)
	Currently employed	186 (33.76)
	Student	30(5.44)
	Missing	73(13.25)
Residence	Gondar town	157 (28.49)
	Outside Gondar town*	394 (71.51)
Disclosure of HIV status	Disclosed	235(42.65)
	Not disclosed	256 (46.46)
	Missing	60 (10.89)
Tuberculosis status	Yes	220(39.93)
	No	331(60.07)
CD4 cells/µL	Median (IQR)	68(41–100)
	<100	292(52.99)
	100–200	178(32.30)
	201–300	48(8.71)
	>300	33(5.99)
Baseline functional status	Functional	169 (30.67)
	Ambulatory	191 (34.66)
	Bed ridden	166 (30.13)
	Missing	25 (4.54)
Baseline WHO stage	Stage I & II	336 (61.20)
	Stage III & IV	213(38.80)

* Residence outside Gondar ∼50 KM average radius. IQR-Inter Quartile Range.

### Outcomes and Reasons of LTF

Among 551 patients who were lost from follow up, 486(88.20%) were successfully tracked and the remaining 65(11.80%) were unable to be tracked (Table 2). For those tracked, death accounted for 233 (48%) of the losses. The non-death losses, (253, 52.06%), were due to relocation to another ART program as self transfer-outs (47%) and stopping ART due to different reasons (53%).

**Table 2:** Outcomes of patients tracked- for LTF.

Outcomes of Tracking		Number (%)
Outcome of tracking(N = 551)	Successfully tracked	486 (88.20)
	Unable to be tracked	65 (11.80)
Outcomes of LTF (n = 486)	Dead	233(47.94)
	Non-dead	253(52.06)
Outcomes for non-dead losses (n = 253)	Stopped ART	135(53.36)
	Self-transfer	118(46.64)

ART- Antiretroviral therap. TO- Transfer-out. LTF-loss to follow-up.

The most common reasons mentioned for not returning to the ART Clinic were: preference of traditional medicine and/or holy water instead of ART (55.56%), perceived improved (30.37%) or deteriorated (25.19%) health status, financial problem (22.22%), and stigma and social problems (9.63%). On the other hand, reasons for the unsuccessful tracking include incorrect, missing or change of addresses (Table 3).

**Table 3:** Reasons for patients stopped ART and not tracked.

Reasons of LTF	Number (%)
**Reason for stopping ART (N = 135)** *	
Preferred traditional medicine and/or holly water	75(55.56)
Improved health	41(30.37)
Deteriorating health	34(25,19)
Financial problem	30(22.22)
Stigma & social problem	13(9.63)
**Reasons for not tracking (N = 65)**	
Incorrect address	43(66.15)
Missing address	16(24.62)
Relocated	6(9.23)

* More than one answer. ART- Antiretroviral Therapy. LTF- Loss to follow-up.

### Characteristics of Death and Non-death Losses

**Figure 2:**
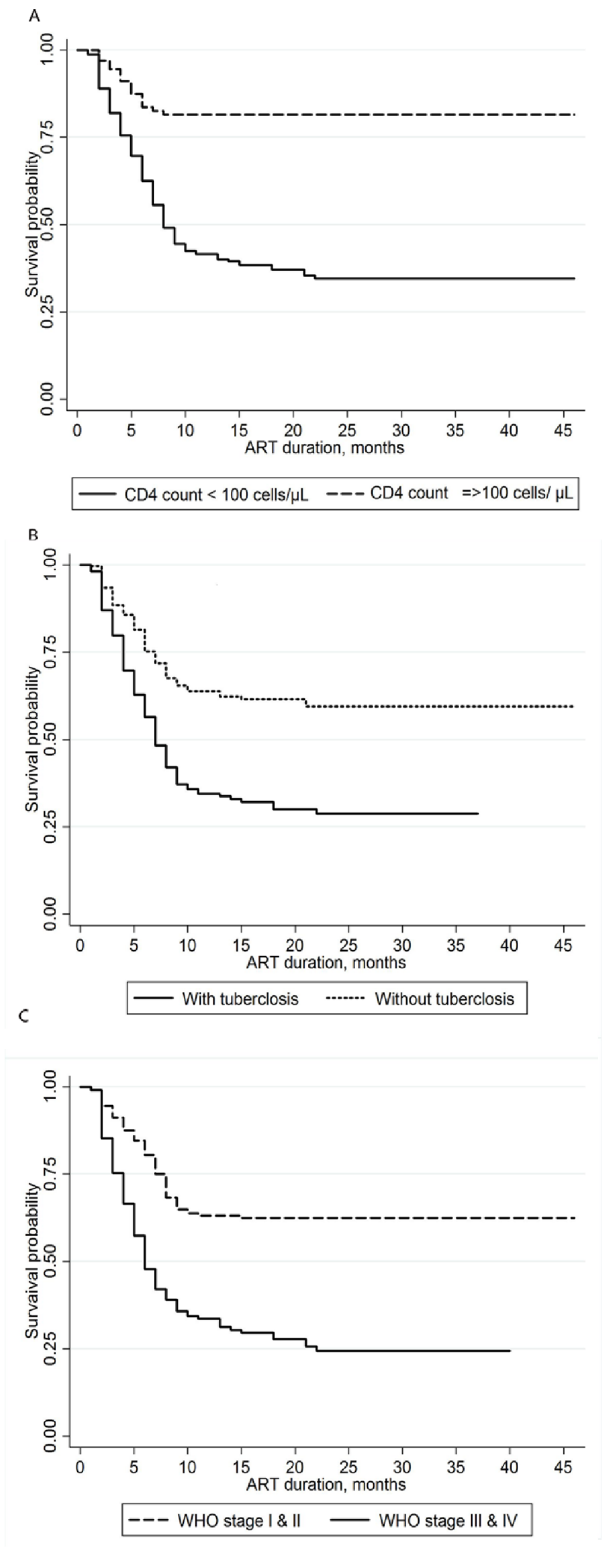
Kaplan-Meier Estimate of Mortality among LTF by baseline clinical characteristics: CD4 cell count (*A*) WHO clinical stage (*B*) and Tuberculosis status (*C*).

The overall incidence of mortality among LTF was 37.74 per 100 person-years (95% CI: 33.18–42.92). The rate of mortality in the first six months was 72.12 per 100 person-years (95% CI: 61.80–84.24) but this sharply decreased after 12 months of follow-up to 7.92 per 100 person-years (95% CI: 4.44–14.4). Mortality in patients with CD4 count <100 cells/µl, WHO stage III and IV, and the presence of tuberculosis at ART initiation was 47.66 (95% CI: 41.65–54.55), 60.40(95% CI: 51.02–71.50) and 54.61(95% CI: 46.01–64.80) per 100 person years, respectively. Kaplan-Meier analyses also showed that the probability of program loss due to death was strongly associated with baseline clinical characteristics (Figure 2), such as baseline CD4 cell count (A), WHO stage of disease (B), and the presence of tuberculosis infection (C), unlike the non-death losses.

Cox proportional hazards model demonstrated that female sex and baseline clinical characteristics such as presence of tuberculosis infection at ART initiation, functional status (both ambulatory and bed ridden); CD4 cell count <100 cells/µL, and WHO stage III and IV were strongly associated with mortality. On the other hand, male sex, bedridden functional status and residence outside Gondar town were significantly associated with non-death losses. Where as, the presence of tuberculosis infection at ART initiation was significantly associated with both death and non-death losses (Table 4).

**Table 4:** Cox-regression model for risk factors for LTF due to death and non-death reasons.

Characteristics	Subcategory	LTF due to Death	LTF due to Non-death
Age		1.01(1.00–.03)	1.00(0.98–0.011)
Gender	Male	0.65(0.49–0.84)	1.73(1.31–2.30)
	Female	1.00	1.00
Place of residence	Gondar town	1.00	1.00
	Outside Gondar town	0.80(0.59–1.07)	0.70(0.52–0.94)
Functional status	Functional	1.00	1.00
	Ambulatory	2.85(1.78–4.56)	0.82(0.62–1.09)
	Bed ridden	5.28(3.35–8.33)	0.55(0.37–0.82)
CD4 cells/µL	<100	2.62(1.64–4.18)	0.95(0.72–1.27)
	≥100	1.00	1.00
WHO clinical stage	Stage I & II	1.00	1.00
	Stage III & IV	2.37(1.80–3.14)	0.96(0.71–1.31)
TB at ART initiation	Yes	1.59(1.21–.2.08)	0.68(0.51–0.92)
	No	1.00	1.00

ART- Antiretroviral Therapy. LTF- Loss to follow-up. WHO-World Health Organization. TB-Tuberculosis.

**Figure 3:**
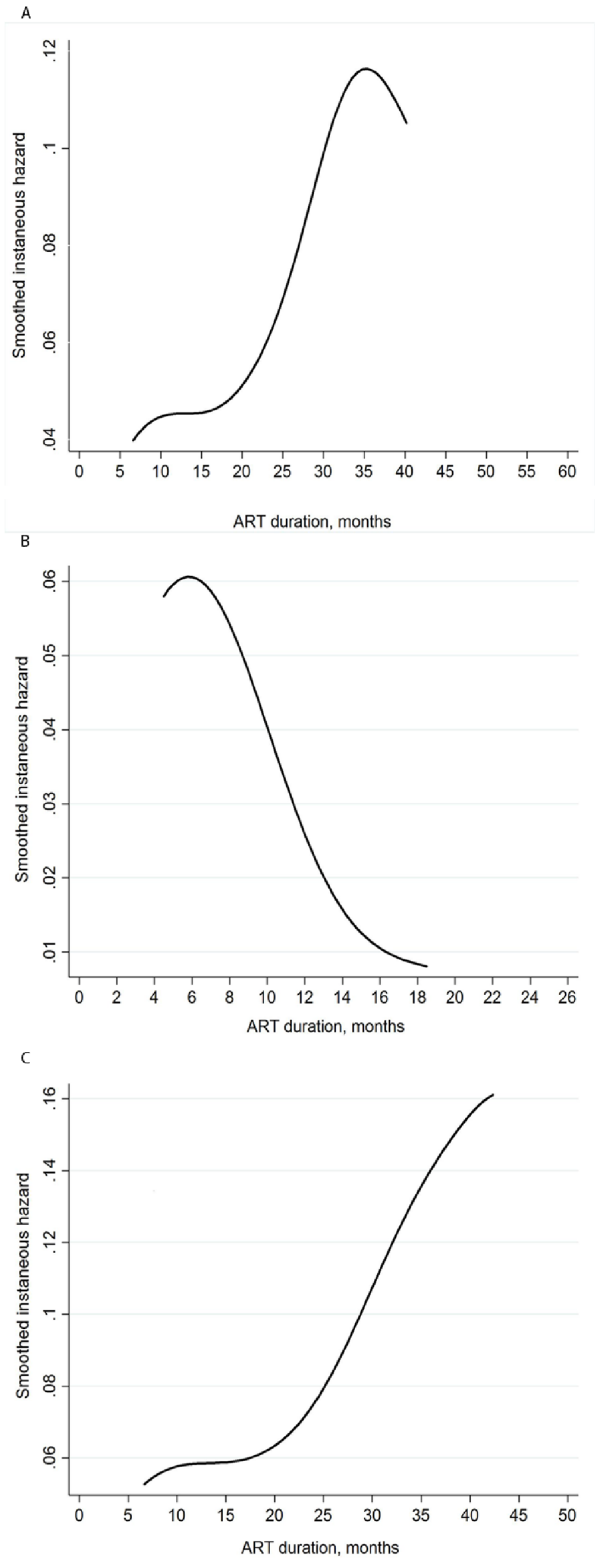
Smoothed hazard estimates for total losses to program (A), death (B), and non-death losses to program(C).

The risk of loss from the program changed markedly during follow-up of patients receiving ART. Risk of death had 3 distinct phases (Figure 3): high in the initial months of ART– but steeply decreasing risk thereafter (*A*) – and a very low risk of death after 1 year of ART (*B*). In contrast, the risk of program loss due to non-death causes (stopping ART and self transfer outs) was low initially, then relatively constant beyond the first year (*C*).

## Discussion

This study clearly demonstrated the characteristics and outcomes of patients LTF in northwest Ethiopia. Overall, losses due to death are highest in the first year while losses due to non-death reasons are highest then after. The study also showed close to half (48%) of the LTF were dead. Preference for traditional medicine and/or holy water instead of ART was the commonest reason for not returning to the ART Clinic in patients with LTF.

A high risk of death in the first few months after starting ART is characteristic of resource-limited settings where most patients start therapy late with advanced disease [7], [14], [15]. However, mortality in this study is substantially higher than the mortality commonly reported in the first year of ART based on routinely recorded deaths [1], [5], [16], [17]. Our study can thus be used to correct estimates of mortality derived without complete tracking of losses to follow up [5], [18]–[20].

In contrast, non-death losses are heterogeneous in this setting. Our finding suggested that in those patients who stopped ART, the risk of losses to the program remain constant after ∼1 year of treatment. This finding is consistent with similar studies [21] except differences in the definition of non-death losses. The effectiveness of ART beyond one year is likely to relate to issues of long-term patient retention rather than death. As patients remain healthy on long-term medication, their motivation to continue treatment in the longer term may diminish. LTF patients who were self-transferred in this program are also substantially higher. In programs with high rates of LTF, those LTF might thus include a sizeable group of low-risk patients who self-transfer out to another program, for example, because of a more convenient location of the new clinic or to avoid stigma or due to work-related reasons [7]. Because the number of patients who are physically well and are receiving long-term medication is increasing, the number of patients moving out of an area for social or economic reasons may continue to increase. Patients might have requested preconditions for official transfer by the health care provider. Further research should understand why patients tempted to self transfer. This finding emphasizes the importance of systems within national ART programs that can ensure the continuity of care for the highly mobile populations. It is important to address patients’ needs to transfer to other treatment centers by providing referral on request without any pre-condition.

The most common reasons mentioned for not returning to the ART Clinic was preference for traditional medicine and/or holy water, instead of ART. Previous studies have found that HIV-infected patients who distrust their health care providers are more likely to use complementary and alternative medicine (CAM) as a substitute for conventional HIV therapy [22]. In addition, HIV-infected patients with a greater desire for medical information and a negative attitude toward the effectiveness of antiretrovirals are more likely to use CAM with potential for adverse effects [23]. Retaining patients who have treatment dilemmas requires HIV clinical personnel to work with religious personnel to provide a model of faith and healing that precludes an “either/or” situation between ART and holy water [24]. Focus should also be placed on sensitizing spiritual leaders to the need for patient adherence to ART concomitant to any faith-based healing practices. Perceived improved or deteriorated health status by the patient is also another reason for not returning to care because of poor understanding of the chronic nature of the disease and the need for continued, life-long ART [7]. Our study also showed that the commonest reasons for unsuccessful tracking were incorrect, missing or change of addresses, which is consistent with reports from similar settings in Ethiopia [16]. Appropriate system to track patients should be a priority in the context of scaling ART. But these issues may not have received sufficient attention from the governmental and nongovernmental organizations driving the scale-up of ART in resource limited settings [25].

Our finding showed that advanced disease stage was a strong predictor of early death is inline with other studies in sub-Saharan Africa [14], [21]. Thirty nine percent of the patients were in Stage III and IV. This finding is different from findings from many other cohort profiles in Ethiopia where more than 50% of the patients are in Stages III and IV combined [26]. This difference might be smaller sample size in our study. We also observed, like other studies in similar settings, that tuberculosis infection at ART initiation was a strong predictor of death and non-death losses [27], [28]. Males were more likely to contribute for death and non death losses [21]. Place of residence outside the Gondar town where the treatment was initiated was associated only with non-death losses. Distance between patients’ residence and the ART Clinic is known to contribute to LTF [29]. Moreover, financial problem for transportation and other related expenses were some of the reasons for stopping medication, as reported in previous studies 7,30.

Some limitations of our study are worth mentioning. Firstly, the study was based in a single ART treatment center so may not be generalisable to a wider context. Secondly, the study was limited to characterizing the outcomes of LTF by physical tracking. Whether the tracking contributed to the re-engagement of patient LTF was not assessed. Future studies should determine the effects of patient tracking on estimates of LTF, mortality and retention in ART programs. Thirdly, the outcome of self-transferred patients was not determined in this study. This might have affected the true estimate of the outcome of LTF. Finally, the study didn’t investigate how and where LTF patients died. Further research should be directed to determine the cause and place of death of patients LTF.

In conclusion, death accounts for about half of the LTF. Most deaths occur in the first six months of loss. Preference of alternative therapies such as traditional medicine and holy water are another major reason for loss to follow up. Early tracking mechanisms are necessary to prevent death. It is also important to address patients’ needs to transfer to other treatment centers by providing referral on request without any pre-condition. Addressing the issues on concurrent use of alternative therapies can also substantially reduce loss to follow up. The findings of the study have implications for patient support and monitoring in ART programs.
